# Oxyphosphate of Zinc as a Filling Material.—Its Abuse

**Published:** 1895-05

**Authors:** J. E. Wilkinson


ARTICLE VI.

### OXYPHOSPHATE OF ZINC AS A FILLING MATERIAL.—ITS ABUSE.

BY J. E. WILKINSON, D. D. S.

Read before the Dental Association of Nova Scotia.

Zinc oxyphosphate, commonly called cement, bone-filling, white filling or composition, has become an essential in operative dentistry. Among its more important uses may be mentioned (1) that of a temporary stopping in young anterior teeth; (2) in deep-seated cavities where pulps are almost exposed (in time often permitting deposits of secondary dentine); (3) in the treatment of nerve-canals to retain the temporary dressing and fill the cavity until considered ready for the permanent filling; (4) as a trial filling over a capping in exposed pulps, and (5) in cases of recent pulpits; (6) as an intermediate in deep cavities under gold or amalgam, both preventing thermal shocks and strengthening frail walls; (7) for inserting crowns and bridges.

Judiciously selected and properly manipulated, in the cases above mentioned, and other conditions and capacities, oxyphosphate has proved to be a great blessing to patients and satisfaction to dentists; but unfortunately, like many other beneficial materials, it has been very sadly misused and abused.

The most common abuse is in being employed as an ordinary filling material in ordinary cases. Its solubility in the fluids of the mouth, the especial tendency to this, in many cases, at the cervical margin and on palatine surfaces, and the consequent uncertainty of its efficient durability, will not allow it a place as a permanent filling. Its use is perhaps most common with foreign dentists having foreign patients, and with itinerant practitioners, the selection appearing to be made owing to the readiness with which the cavity can be prepared and the filling inserted, at the same time commanding a very fair fee, also the unlikelihood of ever seeing the patient again. In some mouths cement will completely dissolve out in about six months, while in others it will continue in good preservation for as many years. Again, in some cases, while the lower part and body of the filling remain intact, the part just under the gum margin has completely dissolved out, forming a most favorable habitat for a colony of microbes which steadily advance in their operations until often the nerve-pulp chamber is penetrated, when an attack of acute pulpitis, or perhaps periostitis, sends the patient to the nearest dentist to find out why a tooth which was filled should ache. Henceforward his or her confidence in dentists is much weakened and accompanied with a constant suspicion. Last fall a lady, the wife of a sea-captain, just before starting on a voyage, had an upper molar extracted. A cavity on the posterior surface had been filled in Calcutta by an American dentist. Fee, four dollars. She wished a filling that would stand, and save the tooth. The material used was cement, which was completely dissolved away at the gingival margin. The nerve was exposed and partly dead, so that under the circumstances the loss of the tooth was necessary.

Many operators neglect to inform their patients that cement is but temporary, and may last only six months. On the other hand, patients who have been warned, very often wilfully neglect obtaining the required attention. How often have we all had patients come to us, especially from the country, with teeth of fairly good quality, but suffering from neglect, who, upon being advised to have them filled, state their intention of letting them go and getting plates some day, because fillings were not of any use, for they had several filled a year or so ago and they all came out, or So-and-So paid $10 or $20 a few years ago for fillings and is now wearing "false teeth." Upon inquiry, we have generally found these to have been the white fillings, and remark that they are only temporary and dissolve out, to which the reply often is, "Yes, that's the way they did, but the dentist didn't tell us that." In this manner good teeth are sacrificed, and the profession in general is injured.

A second abuse of oxyphosphate is in its manipulation, both in mixing and its insertion. By adding too much powder to the fluid at once its quality is lowered. If too stiff when applied to the cavity, it cannot be perfectly adapted, and the filling is granular, rough and defective. If too thin and soft when applied, it is not desirably tough, but crystalline and brittle. Applying to moist cavities, puncturing with small-pointed instruments when nearly hard, and imperfect preparation of cavities, may be mentioned as injurious.

Oxyphosphate is irritating to a pulp partially or almost exposed and its employment as a direct capping is therefore an abuse.

The last abuse of cement to be considered now, is its non-use, referring chiefly to its capacity as an intermediate, in deep-seated cavities, either over a capping where the nerve pulp is partially exposed, or directly applied where the cavity is merely large and deep, for the purpose in either case of lessening thermal shocks, and, perhaps, of strengthening frail walls. How often have all dentists been applied to for relief, by patients suffering from acute periostitis, caused by dead pulps destroyed by irritation caused by large metal fillings where the insertion of non-conducting intermediates might have prevented the trouble. Not only so, but have we not had even to remove metal fillings actually penetrating the nerve-canals?

In speaking of the non-use of cement, we may allude to neglect of young permanent teeth, badly decayed and of very poor quality. A girl of thirteen, whose every tooth, above and below, was decayed, said that she had been taken to a dentist two years before and was told by him that there was no use in filling her teeth. In such a case we know that, if at all possible, the teeth should be preserved until about the age of twenty-one years, when alveolar ridges will be harder and not subject to so marked absorption which is the cause of so great permanent injury, rendering the retention of artificial dentures more difficult, and permanent disfigurement of features. For anterior teeth and often the posterior as well, the material indicated is cement, which should be replaced as often as necessary.—*Dominion Dental Journal.*

